# Notes and News

**Published:** 1903-10-10

**Authors:** 


### Notes and News.

The Hospital Sunday Fund collection this year now equals £63,000.

The Jenner Institute of Preventive Medicine, Chelsea Gardens, will in future be known as the Lister Institute of Preventive Medicine.

The foundation-stone of the City of London Crematorium, which is to cost £7,000, has been laid at Ilford by Mr. R. W. Edwards, chairman of the Sanitary Committee.

A new wing has been opened at the Watford Hospital by the Duchess of Bedford. As far as we know it is the first charitable building established to commemorate King Edward's Coronation. The wing contains two wards of six beds, various offices, dental room and *x*-ray room. The cost has been £4,300.

Professor Ferdinand Hueppe, of Prague, will deliver the Harben Lectures before the Royal Institute of Public Health at King's College, commencing October 8th. The etiology of infectious diseases, hygienic lessons to be derived from the serum treatment, and tuberculosis will comprise the subjects of the lectures.

The German deputation who are visiting England to inspect English arrangements for housing the poor appear to be doing their work very thoroughly. Whilst inspecting the institutions in Liverpool they also paid a visit to the sterilized milk depôt, conducted by the Corporation, for the purpose of checking infant mortality.

A question has arisen as to the exemption of veterinary surgeons from jury service. In Ireland cases can be cited in which veterinary surgeons have been exempted by reason of their calling, but at Brighton, Basingstoke and Worthing requests for exemption have been met with refusals; but it is intimated that the decision might be appealed against. The owners of valuable animals entrusted to the care of veterinary surgeons naturally are interested in this matter.

The plague in Mauritius does not yet show any abatement. The percentage of mortality is still high.

The annual medical service in connection with the Guild of St. Luke will take place at St. Paul's on the 21st instant at 7.30. The Rev. Prebendary Villiers will preach the sermon.

The east block, which constitutes the new extension at the Mount Vernon Hospital for Consumption, will be opened on October 27th by the Marquis of Zetland, President of the Hospital.

The late Miss Ellen Sponge, of Rochester, has bequeathed £1,000 to St. Bartholomew's Hospital of that city, for the establishment of a children's ward to memorialise Mrs. Catherine Spong, her mother.

The Metropolitan Asylums Board are about to substitute permanent buildings for those at present used at the South-Eastern Hospital. The new scheme provides for 488 beds, more accommodation for the staff, and the remodelling of the administrative block. The cost is estimated at £123,000.

The Odontological Society of Great Britain is prepared to receive applications for grants in aid of the furtherance of scientific research in connection with dentistry. Particulars can be obtained from the Honorary Secretary of the Scientific Research Committee of the Odontological Society, 20 Hanover Square, W.

At the last meeting of the London County Council, Lord Monkswell, who presided, remarked in his speech that the Asylum Committee of the Council took their work very seriously, consequently the asylums are kept thoroughly up-to-date, and he alluded to the open-air treatment for consumption which had been established at Bexley.

On Tuesday a public inspection was made of the site of the first so-called Garden City between Hitchin and Baldock, 35 miles from London. The proposal to limit the population, thus providing adequate air space for it, at a cost which shall be within the reach of the class for which it is intended, must meet with approval from the standpoint of commonsense alone.

The medal founded in connection with the Pharmaceutical Society to memorialise the late Mr. Daniel Hanbury, has been awarded to M. Eugéne Collin, of Paris. The medal is awarded every two years by Sir Thomas Hanbury, the founder, for research in the natural history of drugs. In the future Sir Thomas Hanbury will add £50 to the award of the medal to the successful competitor.

The following Entrance Scholarships at Charing Cross Hospital Medical School have been awarded: The Epsom Scholarship, 100 guineas, to Mr. N. G. H. Salmon; the Livingstone Scholarship, 100 guineas, to Mr. R. H. H. Jolly; an Universities Scholarship, 72 guineas, to Mr. W. D. Keyworth. Entrance Scholarships have also been awarded to Mr. P. E. Stibbe, 60 guineas; and to Mr. C. H. F. Atkinson, 30 guineas.

